# Increased Hepatic Insulin Action in Diet-Induced Obese Mice Following Inhibition of Glucosylceramide Synthase

**DOI:** 10.1371/journal.pone.0011239

**Published:** 2010-06-21

**Authors:** Nelson S. Yew, Hongmei Zhao, Eun-Gyoung Hong, I-Huan Wu, Malgorzata Przybylska, Craig Siegel, James A. Shayman, Cynthia M. Arbeeny, Jason K. Kim, Canwen Jiang, Seng H. Cheng

**Affiliations:** 1 Genzyme Corporation, Framingham, Massachusetts, United States of America; 2 Department of Internal Medicine, Yale University School of Medicine, New Haven, Connecticut, United States of America; 3 Department of Endocrinology and Medicine, Hallym University College of Medicine, Seoul, South Korea; 4 Department of Internal Medicine, University of Michigan, Ann Arbor, Michigan, United States of America; 5 Department of Medicine, University of Massachusetts Medical School, Worcester, Massachusetts, United States of America; Hong Kong University, Hong Kong

## Abstract

**Background:**

Obesity is characterized by the accumulation of fat in the liver and other tissues, leading to insulin resistance. We have previously shown that a specific inhibitor of glucosylceramide synthase, which inhibits the initial step in the synthesis of glycosphingolipids (GSLs), improved glucose metabolism and decreased hepatic steatosis in both ob/ob and diet-induced obese (DIO) mice. Here we have determined in the DIO mouse model the efficacy of a related small molecule compound, Genz-112638, which is currently being evaluated clinically for the treatment of Gaucher disease, a lysosomal storage disorder.

**Methodology/Principal Findings:**

DIO mice were treated with the Genz-112638 for 12 to 16 weeks by daily oral gavage. Genz-112638 lowered HbA1c levels and increased glucose tolerance. Whole body adiposity was not affected in normal mice, but decreased in drug-treated obese mice. Drug treatment also significantly lowered liver triglyceride levels and reduced the development of hepatic steatosis. We performed hyperinsulinemic-euglycemic clamps on the DIO mice treated with Genz-112638 and showed that insulin-mediated suppression of hepatic glucose production increased significantly compared to the placebo treated mice, indicating a marked improvement in hepatic insulin sensitivity.

**Conclusions/Significance:**

These results indicate that GSL inhibition in obese mice primarily results in an increase in insulin action in the liver, and suggests that GSLs may have an important role in hepatic insulin resistance in conditions of obesity.

## Introduction

The accumulation of visceral fat in obesity instigates several pathological changes, including chronic low-grade inflammation, steatosis, and insulin resistance [1], [2], [3]. These alterations are closely associated with the development of type 2 diabetes and non-alcoholic fatty liver disease (NAFLD) [4], [5]. With obesity, type 2 diabetes and NAFLD becoming worldwide epidemics, both preventive and therapeutic measures are needed to address these major health care burdens.

A major contributing factor to hyperglycemia in type 2 diabetes is defective regulation of glucose production by the liver [6], [7]. In normal healthy individuals, insulin tightly controls hepatic glucose production directly by suppressing glycogenolysis and gluconeogenesis. Insulin also acts indirectly by inhibiting glucagon secretion from the pancreas, and by suppressing lipolysis and the release of free fatty acids from adipose tissue and gluconeogenic precursors from skeletal muscle, all of which stimulate gluconeogenesis [8]. In obese and diabetic patients, hepatic steatosis results in a failure of insulin action and consequently leads to excessive hepatic glucose production (HGP) and fasting hyperglycemia [6].

We have previously shown that a small molecule inhibitor of glucosylceramide synthase (GCS), the initial and rate-limiting enzyme involved in the biosynthesis of gangliosides and other glycosphingolipids (GSLs), improved glycemic control, decreased insulin resistance, and inhibited the development of hepatic steatosis in several animal models of type 2 diabetes [9], [10]. Aerts et al [11] also obtained similar results using an imino-sugar based inhibitor of GCS. These data pharmacologically validated GSLs as having an important role in insulin signaling and hepatic steatosis, confirming the original observation that transgenic knockout mice lacking ganglioside GM3 and downstream GSLs are resistant to glucose intolerance caused by a high fat diet (HFD) [12], [13]. It is not known how GSLs are affecting insulin signaling, although the current hypothesis is that GSLs within lipid rafts or microdomains may be modulating the activity of various membrane-associated receptors, including the insulin receptor. Also unclear is the primary mode of action of our GCS inhibitors. Therefore, to better understand how our GCS inhibitors are affecting glucose metabolism in different tissues, we have performed hyperinsulinemic-euglycemic clamps in diet-induced obese (DIO) mice that had been treated with our small molecule compounds, and used radio-labeled metabolites to determine the effect of drug treatment on the uptake of glucose into different tissues.

Genz-112638 (eliglustat tartrate) is a small molecule inhibitor of glucosylceramide synthase (GCS) that was originally developed for substrate reduction therapy of Gaucher disease, which is characterized by the accumulation of glucosylceramide (GL1) in the lysosomes of affected individuals [14]. In vitro, Genz-112638 exhibits good potency with an IC50 of ∼24 nM against GCS and no detectable inhibition of α-glucosidases, saccharases, or lysosomal glucocerebrosidase. The compound also has no inhibitory activity against either neutral or acid ceramidase and does not alter cellular ceramide levels either in vitro or in vivo. In rodents, Genz-112638 is rapidly metabolized with a half-life of 15–45 minutes. When administered to a murine model of Gaucher disease by daily oral gavage, the compound decreases GL1 levels in the liver by ∼20% at a dose of 75 mg/kg and by ∼60% at a dose of 150 mg/kg [14]. While Genz-112638 is comparable in activity to Genz-123346, which was used in previous studies [9], [10], Genz-112638 has a more favorable pharmacokinetic and pharmacodynamic profile for use in humans. In addition, unlike Genz-123346, Genz-112638 contains a natural ceramide structure, i.e. an even number of carbons in its acyl chain. Therefore, Genz-112638 was chosen for use in clinical trials for Gaucher disease, and it was of interest to also evaluate this compound preclinically in animal models of type 2 diabetes. The results suggest that inhibiting GSLs with Genz-112638 partially corrects the impaired regulation of HGP in obese mice.

## Results

### Effect of Genz-112638 on liver sphingolipid levels

**Figure 1:**
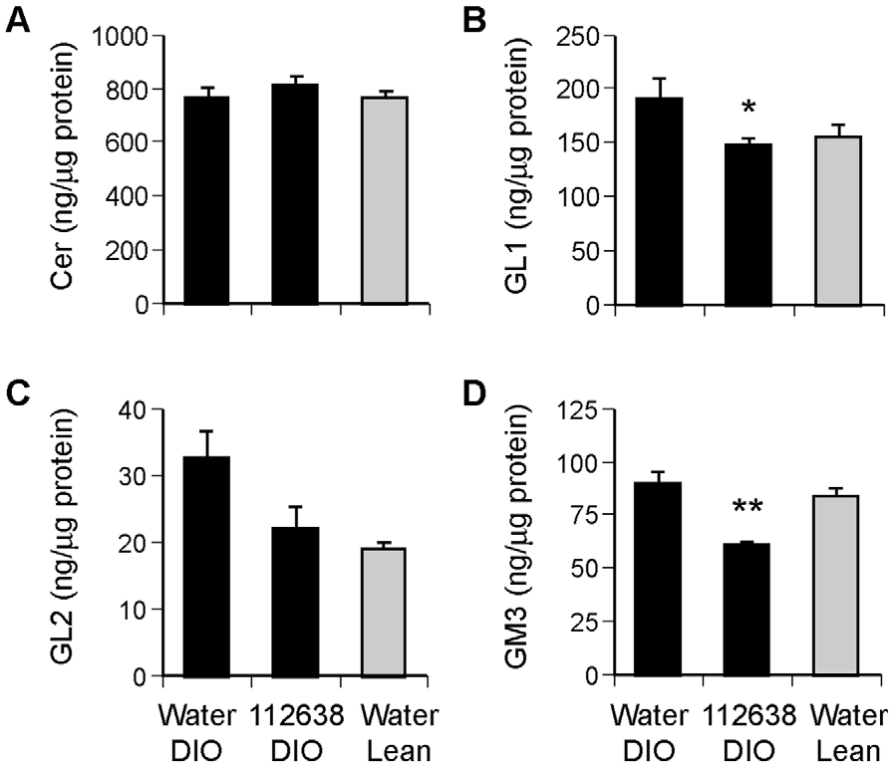
Sphingolipid levels in the livers of DIO mice after treatment with Genz-112638. C57BL/6 mice were fed a normal diet (Lean) or a HFD (DIO) for 8 weeks. The DIO mice were then treated with Genz-112638 or placebo (water) by twice daily oral gavage (120 mg/kg/day) for 22 weeks, while remaining on the HFD. Sphingolipid levels were determined by high performance liquid chromatography – tandem mass spectrometry (see Materials and Methods). Data shown as mean ± SEM (n = 6–9 mice per group). *P<0.05, **P<0.01 Genz-112638 DIO vs. Water DIO.

The short half-life and rapid metabolism of Genz-112638 in rodents necessitates daily or twice daily dosing to achieve sufficient exposure in tissues over time. We determined that a dose of 120–125 mg/kg/day was well-tolerated and effectively lowered GL1 and GM3 levels when administered by daily oral gavage. A representative study is shown in Fig. 1. Mice fed a high fat diet (DIO) or normal chow (Lean) were treated with Genz-112638 at 120 mg/kg/day for 22 weeks. Liver ceramide levels were unchanged in the drug treated animals, while liver GL1 and GM3 levels were decreased by approximately 20–30% (Figs. 1A, B, and D). The unaltered ceramide levels in the animals treated with Genz-112638 was not unexpected, since the drug does not block ceramide synthesis. While ceramide could possibly accumulate due to inhibiting the conversion of ceramide to GL1 and other GSLs, this was not observed. Liver GL2, which is expressed at low levels, trended lower but was not decreased significantly after drug treatment (Fig. 1C). The duration of drug treatment in Fig. 1 was longer than in subsequent studies, which was typically 12–16 weeks. However, we have observed that the decrease in lipid levels stabilizes within one week of dosing, and further lipid lowering was not observed with longer treatment (data not shown).

### Effect of Genz-112638 on food consumption and body weight

**Figure 2:**
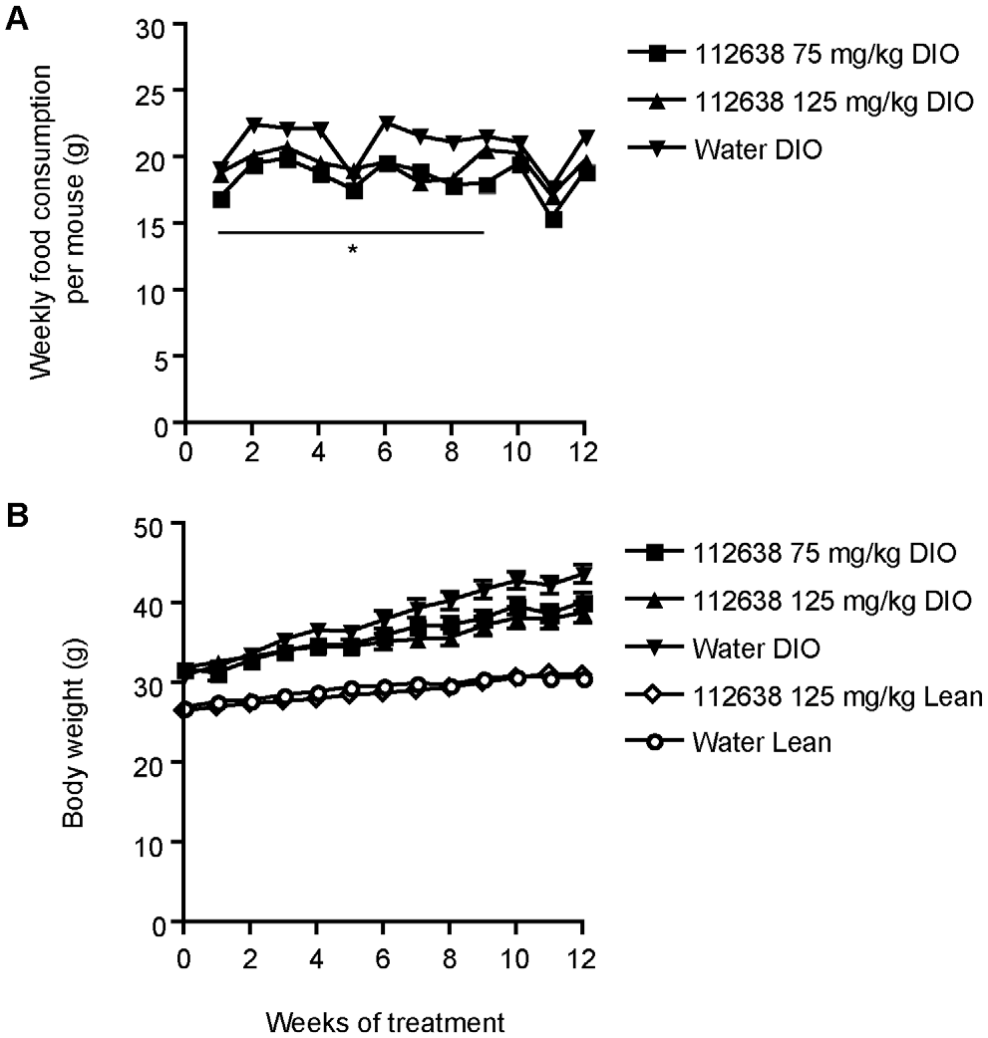
Effect of Genz-112638 on A) food consumption and B) body weight. C57BL/6 mice were fed a normal diet (Lean) or a HFD (DIO) for 8 weeks. The DIO or Lean mice were then treated with Genz-112638 or placebo (water) by daily oral gavage (125 mg/kg/day) for 12 weeks, while remaining on their respective diets. Data shown as mean ± SEM (n = 7–14 mice per group). *P<0.05 Genz-112638 DIO (both doses) vs. Water DIO, weeks 0–9. There was no significant difference in body weights between the Water DIO and drug treated groups.

To evaluate Genz-112638 in the DIO mouse, we placed normal C57BL/6 mice on the high fat diet (HFD, 45% kcal from fat) beginning at 5 weeks of age. After 8 weeks on the diet, the mice were grouped so as to have comparable glucose and insulin levels and body weights. We then administered either Genz-112638 or placebo (water) to the mice by daily oral gavage. In the DIO mice, drug treatment at either 75 or 125 mg/kg/day resulted in a slight reduction in food consumption compared to the animals treated with placebo (water)(Fig. 2A). This decrease was significant (P<0.05), although the difference between the groups became less at the later timepoints (e.g. weeks 10–12). While there appeared to be a corresponding small reduction in weight gain in the drug treated versus water treated animals (Fig. 2B), this difference was not statistically significant. In the Lean mice, the weight of food consumed was considerably higher (>30 g/week) due to the lower caloric density of normal chow, but there was no substantial difference in food consumption between the drug treated versus water treated animals (data not shown). The body weights of the drug versus placebo treated Lean mice were similar (Fig. 2B). The results indicate that treatment with Genz-112638 did not significantly alter body weight gain in either the DIO or Lean mice.

### Genz-112638 lowered hemoglobin A1c levels and improved glucose tolerance in DIO mice

**Figure 3:**
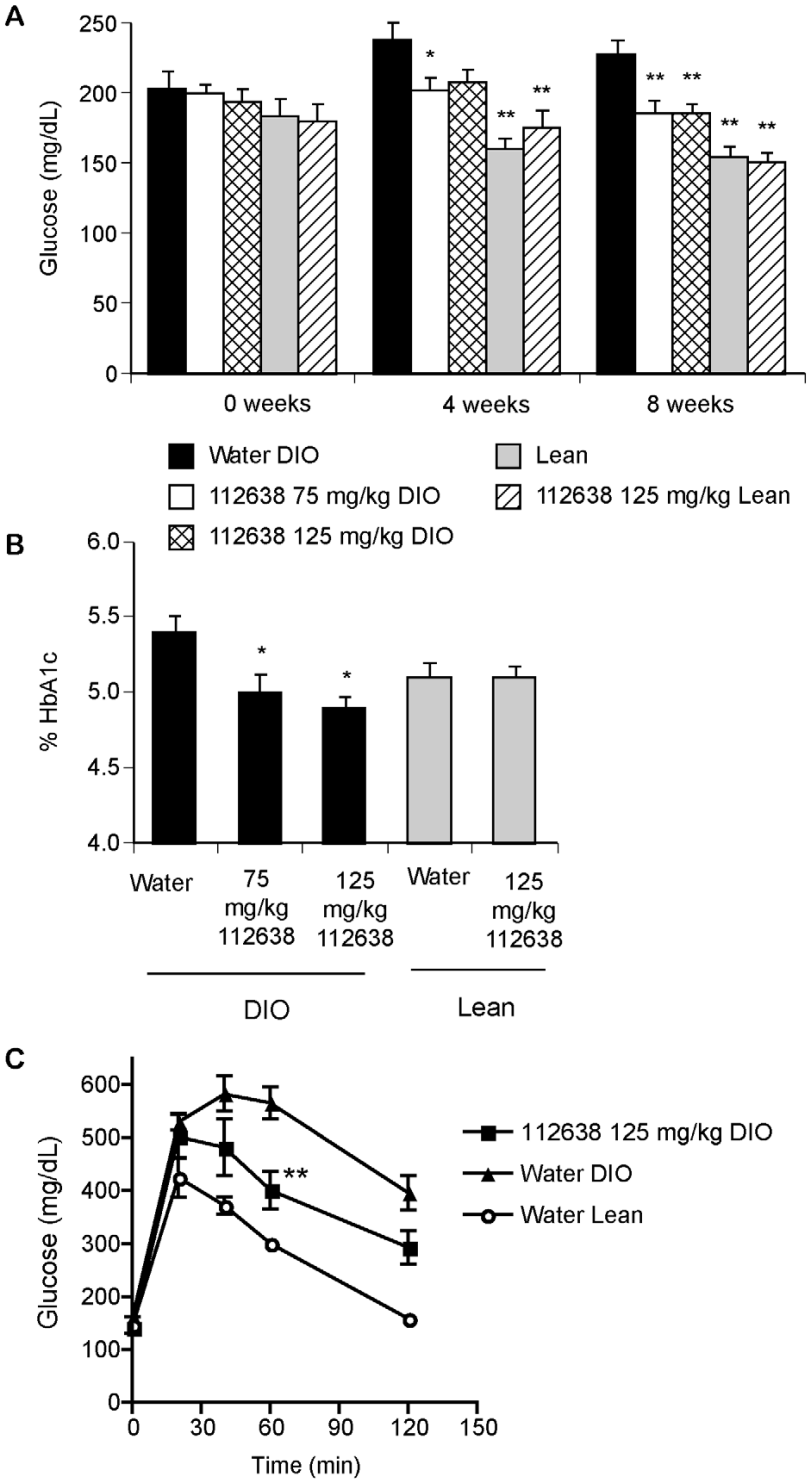
Non-fasting glucose levels, hemoglobin A1c (HbA1c), and glucose tolerance test of DIO mice treated with Genz-112638. A) DIO mice were treated with Genz-112638 or placebo (water) by daily oral gavage at 75 or 125 mg/kg/day and non-fasting glucose levels were measured after 0, 4, and 8 weeks of treatment. B) HbA1c levels after 12 weeks of treatment. C) Glucose tolerance test. DIO mice that had been treated with Genz-112638 for 10 weeks were fasted overnight and then injected intraperitoneally with glucose (2g/kg) the following morning. Glucose levels were measured 0, 20, 40, 60, and 120 minutes post-injection. Data shown as mean ± SEM (n = 6–16 mice per group). *P<0.05, **P<0.01 vs. Water DIO.

We then determined the effect of Genz-112638 on glucose levels and glucose tolerance in the diet-induce obese (DIO) mouse. C57BL/6 mice were placed on either the HFD or normal rodent chow for 8 weeks. The high fat fed (DIO) or chow fed (Lean) mice were then orally gavaged daily with Genz-112638 at 75 or 125 mg/kg/day for 12 weeks while remaining on their respective diets. There was no difference in non-fasting glucose levels between the DIO and Lean mice at the initiation of treatment (0 week time point) when the mice had been on the HFD for only 8 weeks. The DIO mice became mildly hyperglycemic after an additional 4 weeks on the HFD, and treatment with Genz-112638 resulted in a small but significant decrease in glucose levels (P<0.01 at both the 75 mg/kg and 125 mg/kg doses after 8 weeks of treatment)(Fig. 3A). Drug treatment more consistently lowered hemoglobin A1c (HbA1c) to levels comparable to those of the Lean mice (Fig. 3B). Genz-112638 treatment of Lean mice had no effect on HbA1c levels. The DIO mice treated with drug also exhibited improved glucose tolerance (Fig. 3C). These data are consistent with what we previously observed with a related compound (Genz-123346) [9], and confirm that GCS inhibition improves glycemic control in the DIO mouse model.

### Genz-112638 reduced whole body fat mass in DIO mice

**Figure 4:**
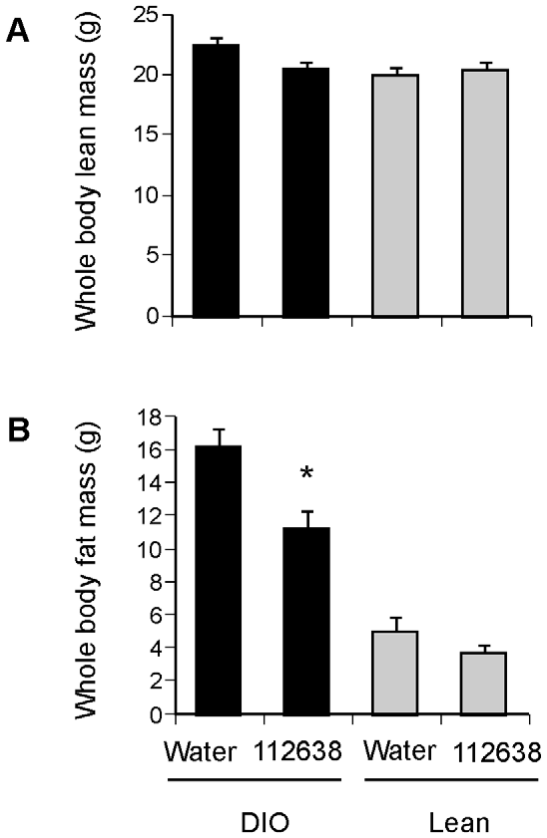
Effect of Genz-112638 on A) whole body fat mass, and B) whole body lean mass. C57BL/6 mice were fed a normal diet (Lean) or a HFD (DIO) for 8 weeks. The mice were then treated with Genz-112638 or placebo (water) by daily oral gavage (125 mg/kg/day) for 12 weeks, while remaining on their respective diets. Fat and lean mass were measured by proton magnetic resonance spectroscopy (^1^H-MRS) on awake mice. Data shown as mean ± SEM (n = 10 mice per group). *P<0.05 Genz-112638 DIO vs. Water DIO.

As shown in Figure 2B, we observed that the high fat fed mice treated with Genz-112638 exhibited a small reduction in weight gain over time. Using proton magnetic spectrometry (^1^H-MRS) on awake, unanesthetized mice, we quantitated whole body lean and fat mass for mice that had been treated with Genz-112638 (125 mg/kg) or placebo (water) by daily oral gavage for 12 weeks while on either a normal or HFD. Lean mass was not affected by drug treatment on either diet (Fig. 4A). However, whole body fat mass was significantly reduced in the Genz-112638 treated mice fed the HFD (Fig. 4B).

### Genz-112638 decreased hepatic steatosis in DIO mice

**Figure 5:**
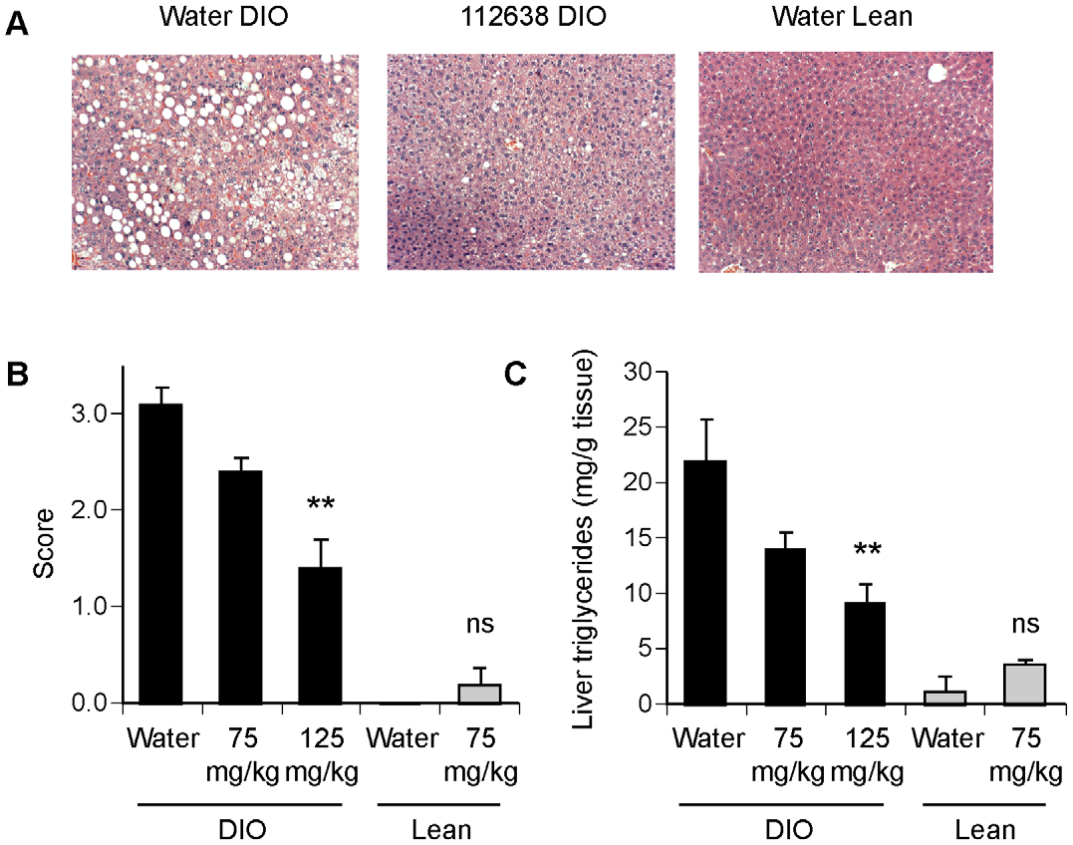
Effect of Genz-112638 on hepatic steatosis. DIO mice were treated with Genz-112638 or placebo (water) by daily oral gavage at 75 or 125 mg/kg/day for 16 weeks. A) Representative liver sections stained with hematoxylin and eosin (H&E). B) Fatty change score of the livers (see Methods). C) Liver triglyceride levels. Data shown as mean ± SEM (n = 6–10 mice per group). **P<0.01 Genz-112638 DIO vs. Water DIO.

We next determined the effect of Genz-112638 on steatosis in the liver that typically develops in mice given a high-fat diet. The livers from DIO mice that had been treated with Genz-112638 (75 or 125 mg/kg) for 16 weeks were sectioned and stained with hematoxylin and eosin or with Oil Red O, which stains neutral lipids. The livers from the placebo (water) treated animals contained numerous vacuoles of variable size as well as cells having a “foamy” appearance (Fig. 5A). In contrast, the livers from mice treated with Genz-112638 contained significantly fewer vacuoles and the vacuoles present were often smaller in size compared to those found in the control livers. The degree of hepatocellular fatty change was scored on a scale of 0 to 4, with 0 signifying no fatty change and 4 signifying marked change with large lipid globules in >75% of hepatocytes. Treatment with Genz-112638 at 125 mg/kg resulted in a significant decrease in the fatty change score from 3.1±0.29 to 1.4±0.18 (P<0.001) (Fig. 5B). This result was confirmed by morphometric analysis, in which the fractional vacuole area of the livers from the drug treated animals was reduced from 0.15±0.04 (Water DIO) to 0.055±0.01 (125 mg/kg Genz-112638 DIO)(P<0.05). In addition, we observed a dose-dependent decrease in liver triglyceride levels (Fig. 3C). These results indicate that Genz-112638 largely prevented the development of hepatic steatosis caused by high fat feeding.

### Effect of Genz-112638 on whole body and hepatic glucose metabolism during hyperinsulinemic-euglycemic clamps in normal and DIO mice

**Figure 6:**
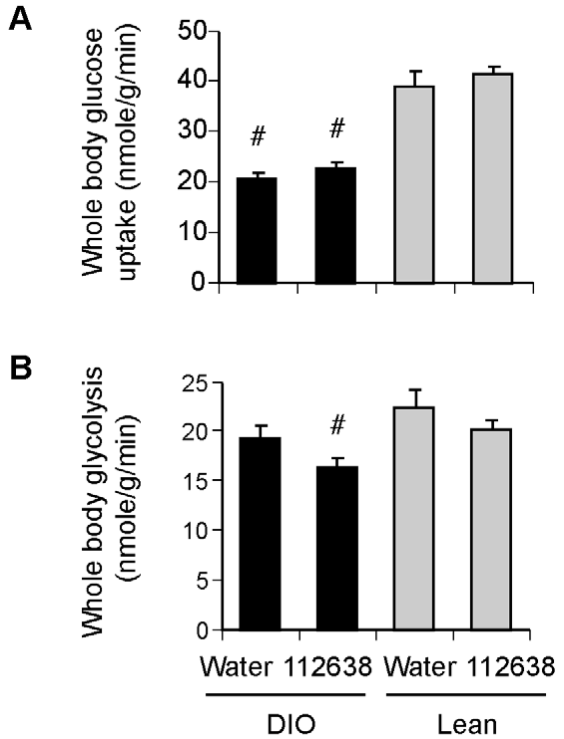
Effect of Genz-112638 on whole body glucose metabolism. Hyperinsulinemic-euglycemic clamps were performed in normal diet (Lean) or HFD (DIO) fed mice treated with Genz-112638 or placebo (water) by daily oral gavage (125 mg/kg/day) for 12 weeks. Animals were continuously infused with [^3^H]glucose throughout the clamps. A) Insulin-stimulated whole body glucose uptake, B) Insulin-stimulated whole body glycolysis. Data shown as mean ± SEM (n = 10 mice per group). #*P*<0.05 vs. Water Lean, *P<0.05 Genz-112638 DIO vs. Water DIO.

To investigate how Genz-112638 is effecting these changes and possibly altering whole body or tissue-specific glucose metabolism, we performed hyperinsulinemic-euglycemic clamps in mice fed either normal chow or a HFD for 12 weeks plus or minus treatment with Genz-112638 (125 mg/kg/day). Insulin-stimulated whole body glucose uptake, which was determined by the ratio of the [^3^H] glucose infusion rate to the specific activity of plasma glucose during the final 30 minutes of the clamp, was significantly decreased in the DIO mice (Fig. 6A). Whole body glycolysis, which was calculated from the rate of increase in plasma ^3^H_2_O concentration during minutes 90–120 of the clamp, was also impaired in the DIO mice as expected. Treatment with Genz-112638 did not significantly increase either insulin-stimulated whole body glucose uptake or whole body glycolysis (Fig. 6A and B). Although somewhat unexpected, the results indicated that drug treatment was unable to cause a measurable increase in overall glucose metabolism, at least at the particular concentration of insulin used during the clamps. Genz-112638 treatment also did not improve glucose uptake or glycogen synthesis in skeletal muscle (data not shown). In accordance with these results, we have not observed any change in the glycogen content of the liver or skeletal muscle as a result of drug treatment (Table 1). However, given the variability in glycogen levels between animals, and the absence of a difference in glycogen content between the Lean and DIO tissues, any significant changes in glycogen levels due to drug treatment was not anticipated.

**Table 1:** Glycogen content of livers and muscles after treatment with Genz-112638.

Group	Liver glycogen (mg/g tissue)	Muscle glycogen (mg/g tissue)
Water DIO	15.7±3.1	1.2±0.2
112638 DIO	16.9±2.4	1.2±0.2
Water Lean	10.0±1.3	1.2±0.2

C57BL/6 mice that were fed a normal diet (Lean) or a HFD (DIO) were treated as described in the legend to Fig. 1. Data shown as mean ± SEM (n = 6–9 mice per group). *P*>0.05 between all groups.

**Figure 7:**
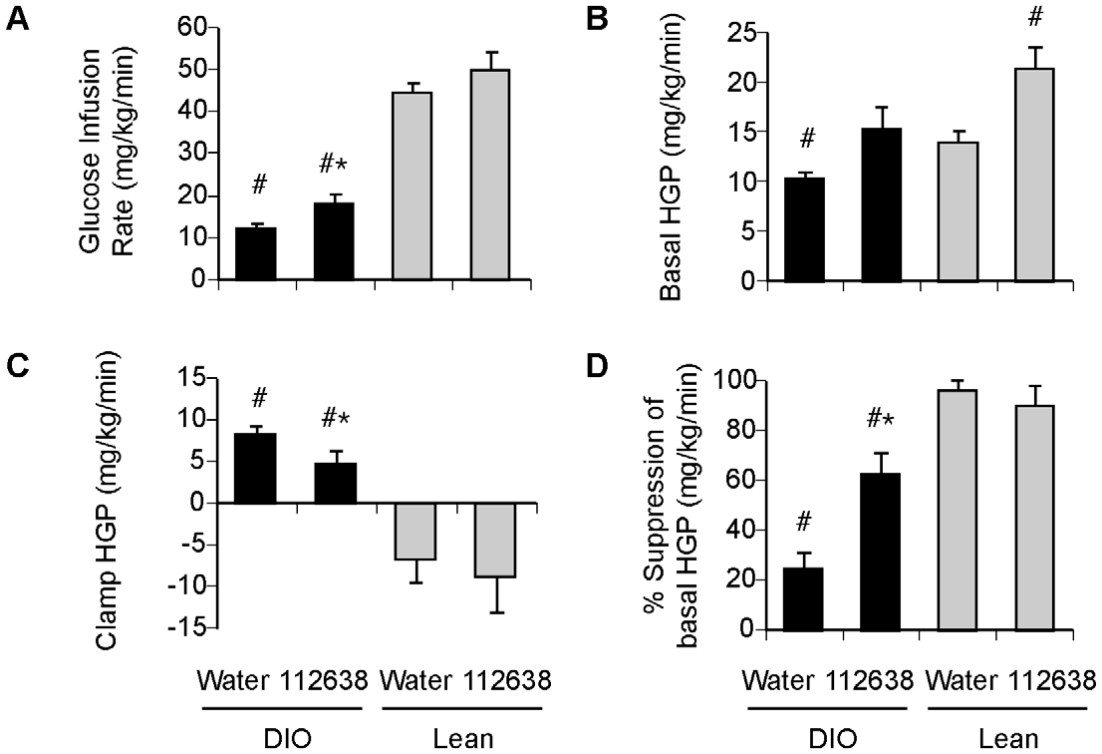
Effect of Genz-112638 on hepatic glucose metabolism. Hyperinsulinemic-euglycemic clamps were performed in normal diet (Lean) or HFD (DIO) fed mice treated with Genz-112638 or placebo (water) by daily oral gavage (125 mg/kg/day) for 12 weeks. Animals were continuously infused with [^3^H]glucose throughout the clamps. A) Steady-state glucose infusion rates, B) Basal HGP, C) Clamp HGP, D) Insulin-mediated percent suppression of basal HGP. Data shown as mean ± SEM (n = 10 mice per group). #*P*<0.05 vs. Water Lean, *P<0.05 Genz-112638 DIO vs. Water DIO.

Treatment with Genz-112638 did result in a modest but statistically significant increase in the steady-state glucose infusion rate needed to maintain euglycemia (Fig. 7A). Prior to the insulin clamp, basal HGP was observed to be higher in both the DIO and Lean mice that received Genz-112638 (Fig. 7B). After applying the 2.5 mU/kg/min insulin clamp, HGP in the Lean mice was essentially zero as expected. The clamp HGP in the DIO mice was only slightly lower than the basal HGP, but decreased to a greater extent in the drug-treated DIO mice (Fig. 7C). Thus, overall Genz-112638 markedly increased the percent suppression of HGP in the high-fat fed mice (Fig. 7D). The results suggest that the basis by which Genz-112638 improved glycemic control in DIO mice may be due in part to increased hepatic insulin sensitivity, thereby improving insulin-mediated suppression of glucose production from the liver.

## Discussion

In these studies we have shown that Genz-112638, an inhibitor of GSL synthesis, lowered HbA1c levels, improved glucose tolerance, decreased whole body adiposity and significantly reduced the development of steatosis in the liver of DIO mice. These data concur with our previously reported results using a chemically related GCS inhibitor, Genz-123346 [9], [10]. To gain further insight into the mechanism underlying these observations, we performed hyperinsulinemic-euglycemic clamps with radioisotope-labeled glucose to determine the effect of drug treatment on whole body and tissue specific glucose metabolism. High fat feeding resulted in defects in both whole body and hepatic glucose metabolism. Genz-112638 improved insulin sensitivity and significantly increased insulin-stimulated suppression of HGP in obese mice. The data suggest that augmented hepatic insulin action may be a primary effect of GSL inhibition in the DIO mouse.

Analysis of liver sphingolipids showed a trend toward increased GL1 and GL2 in DIO mice compared to their lean counterparts, but overall there were no dramatic differences, and ceramide levels were unchanged. This result is in contrast to the reported highly elevated levels of ceramide in ob/ob mice compared to normal mice (>800% increase) and the 20–40% increase in liver ceramide levels in obese Zucker rats [11], [15], [16]. Ceramide has been shown to have a critical role in insulin resistance [17]. Inhibiting serine palmitoyltransferase, the initial rate-limiting step in sphingolipid synthesis, with myriocin lowered ceramide levels, improved glucose tolerance, increased insulin sensitivity, and decreased hepatic steatosis in diabetic rodent models [16], [18]. The much less severe phenotype of the DIO mouse compared to the genetically-induced obese animals may account for the absence of increased ceramide synthesis and accumulation, at least in the liver. Our data suggest that the hepatic insulin resistance and steatosis that develops in the DIO mouse is not due to increased hepatic ceramide. Conversely, the increased insulin sensitivity and decreased steatosis in the liver after treatment with Genz-112638 is not due to lowering ceramide levels, but rather may be a consequence of lowering GSL levels. Notably, myriocin has been shown to decrease not only ceramide but also GSLs, and part of the phenotypic improvements observed with myriocin may be partly attributed to the decrease in GSLs rather than just the decrease in ceramide alone [19].

It remains to be determined whether drug treatment is affecting the direct or indirect actions of insulin on HGP, or both. Genz-112638 may directly increase hepatic insulin receptor (IR) signaling, since GSLs within lipid rafts are known to modulate the activity of the IR and other membrane associated receptors [20], [21], [22]. We and others [9], [11] have shown that GCS inhibitors activate IR autophosphorylation and downstream effectors both in vitro and in vivo, and recently Bijl et al. [23] have shown improved hepatic insulin signaling, as evidenced by increased Akt and mTOR phosphorylation, using the iminosugar-based GCS inhibitor N-(5′-adamantane-1′-yl-methoxy)-pentyl-1-deoxynojirimycin (AMP-DNM) in ob/ob mice. In preliminary studies we have observed an apparent increase in IR activation in the livers of DIO mice that received Genz-112638 (data not shown), although the variability between animals and the fact that IR signaling in DIO mice livers is not significantly impaired compared to that observed in ob/ob mice has precluded us from a definitive result.

Alternatively, one can speculate that Genz-112638 may alter the known indirect effects of insulin on HGP through actions in the pancreas, skeletal muscle, or adipose tissue. For example, Genz-112638 may augment the ability of insulin to inhibit glucagon secretion from pancreatic α-cells, or enhance the ability of insulin to inhibit the release of gluconeogenic precursors from skeletal muscle and non-esterified fatty acids from adipose tissue, thereby reducing hepatic gluconeogenesis [8]. Previous studies using both imino-sugar based GCS inhibitors and ceramide-based inhibitors have demonstrated increased insulin signaling in both skeletal muscle and adipose tissue of DIO and ob/ob mice [9], [24]. The ability of Genz-112638 to decrease whole body adiposity in DIO mice suggests a role for indirect alterations of HGP through improvements in overall lipid metabolism. The decrease in fat mass may be due to an increase in fatty acid oxidation, although we were not able to observe any increase in energy expenditure or oxygen consumption when ob/ob mice were treated with a related GCS inhibitor (Genz-123346, data not shown). The effect of Genz-112638 on hepatic inflammation also needs to be evaluated. Despite the presence of steatotic vacuoles, the degree of inflammation as assessed histologically was considered within the normal limits of wild type mice and was not affected by drug treatment. However, we have reported previously a decrease in TNF-alpha in the livers of ob/ob mice, and reduced ALT levels in DIO mice treated with Genz-123346 [10]. Overall, it is likely that Genz-112638 is both having direct and indirect effects on regulating HGP, and additional studies are needed to better understand the how lowering GSLs impacts both the liver and peripheral tissues to regulate hepatic glucose output.

In conclusion, Genz-112638 has been shown to at least partially address the insulin resistance, excessive glucose production, and steatosis in the liver that are hallmarks of obesity and type 2 diabetes. While a number of available and prospective therapies target hepatic dysregulation either directly or indirectly, including metformin, glucagon-like peptide-1 (GLP-1) analogs, glucagon receptor antagonists, thiazolidinediones, and enzyme inhibitors of gluconeogenesis [25], the use of inhibitors of GSL synthesis is mechanistically quite distinct from these approaches. Therefore, Genz-112638 or related inhibitors may represent a promising and unique therapeutic strategy to correct hepatic insulin resistance and the associated metabolic disorders that result.

## Materials and Methods

### Animals

Male C57BL/6 mice were purchased from the Jackson Laboratory (Bar Harbor, ME). Diet-induced obese (DIO) mice were generated by placing the mice, beginning at 5 weeks of age, on a high fat diet (45 kcal% fat) (D12451, Research Diets, Inc., New Brunswick, NJ) for 8 weeks. Control animals were placed on normal rodent chow. The mice remained on either the high fat or chow diet during the drug-treatment phase. Animals were cared for in an AAALAC accredited facility in accordance with the guidelines established by the National Research Council.

### Administration of drug

Genz-112638 is a small molecule inhibitor of glucosylceramide synthase and is an analog of 1-phenyl-2-decanoylamino-3-morpholino-1-propanol (PDMP) [26]. The synthesis, structure, and in vitro activity of Genz-112638 have been described previously [14]. Animals were orally gavaged once a day with a solution of Genz-112638 in water, or water alone (control group).

### Measurement of sphingolipid levels

Liver tissue (15–25 mg) was cut and weighed directly into glass vials. The tissue was homogenized in a methanol extraction buffer with a hand-held tissue tearor until the tissue was fully dispersed. The vial was then placed in a water bath sonicator for 10 min, vortexed briefly, and the homogenate transferred to a 15 mL centrifuge tube. The tube was centrifuged at 4000 rpm for 5 min to pellet debris. The supernatant was added to vials containing dried internal standards and vortexed to help incorporate the internal standards into solution. Samples were analyzed on a system consisting of an HTC PAL autosampler (CTC Analytics AG, Zwingen, Switzerland), Agilent 1200 HPLC, and API-4000 mass spectrometer (Applied Biosystems, Foster City, CA). During analysis, samples were stored at 9°C in the autosampler cool stack. The HPLC was run in isocratic mode with a normal-phase silica column, and MS/MS was performed in the MRM (multiple reaction monitoring) mode.

### Blood analysis

Blood samples were collected by either tail vein nick (glucose) or retroorbital plexus puncture (HbA1c). Non-fasting glucose levels were measured using an Accu-Chek Compact® Meter (Roche Diagnostics, Indianapolis, IN). HbA1c levels were measured using A1cNow® Monitors (Metrika, Inc., Sunnyvale, CA).

### Whole body composition

Measurement of whole body fat and lean masses was performed using a quantitative magnetic resonance body composition analyzer (Bruker Mini-Spec Analyzer, Echo Medical Systems, Houston, TX).

### Histopathology

Livers were fixed in 10% neutral buffered formalin and embedded in paraffin. Sections were then stained with hematoxylin-eosin. To visualize neutral lipids, livers were frozen in Tissue-Tek O.C.T. compound (Sakura Finetek USA, Inc., Torrance, CA) and sections were stained with Oil Red O. The sections were examined by a board-certified veterinary pathologist and scored on a scale of 0–4 for the degree of hepatocellular fatty change as follows: 0, no significant fatty change; 1, minimal fatty change with minute Oil Red O positive globules in <50% of hepatocytes; 2, mild fatty change with small Oil Red O positive globules present in 20–100% of hepatocytes and with <10% containing large vacuoles; 3, moderate fatty change with Oil Red O positive globules of variable size in >75% of hepatocytes and cytoplasmic detail obscured in <25% of hepatocytes; 4, marked fatty change with Oil Red O positive globules of variable size and including many large globules filling or expanding the cytoplasm of >75% of hepatocytes; cytoplasmic detail is obscured in >50% of hepatocytes; neutrophil infiltrates associated with cell rupture and leakage of lipid.

### Quantification of hepatic steatosis and triglycerides

Hepatic steatosis was quantitated by morphometric analysis as described [10]. Briefly, ten to eighteen independent fields were acquired from each liver section using a microscope set up for bright field illumination using a 20× objective lens. A program was assembled and executed using MetaMorph image analysis software (Molecular Devices, Sunnyvale, CA), which automatically identified areas of each image occupied by total tissue, foamy cells, and vacuoles based upon size, color, and optical density. To measure triglyceride levels, livers were homogenized in water and total lipids were extracted with methanol∶chloroform (4∶3.3). The extracted lipids were dried in glass vials and then resuspended in dimethylsulfoxide. Triglycerides were measured using the Serum Triglyceride Determination kit (TR0100, Sigma-Aldrich, St. Louis, MO).

### Hyperinsulinemic-euglycemic clamp

This study was performed at the Yale Mouse Metabolic Phenotyping Center and all procedures were approved by the Yale University Animal Care and Use Committee. From a cohort (n = 120) of mice given the high fat diet for 8 weeks, the upper 50% with respect to body weight and whole body fat mass gain were initially selected. Of these mice, 24 with the highest glucose and insulin levels were selected for the study. The 24 DIO mice and 24 lean mice were then treated with Genz-112638 or placebo (water) by daily oral gavage (125 mg/kg) for 12 weeks, while remaining on their respective diets. At 3–4 days prior to the day of the clamp, indwelling catheters were inserted into the right jugular vein. After an overnight fast (∼15 h), a 2 h hyperinsulinemic-euglycemic clamp was performed as described previously [27]. Briefly, human insulin (Humulin, Eli Lilly, Indianapolis, IN) was infused continuously to raise plasma insulin levels, and glucose was infused at variable rates to maintain euglycemia. Basal and insulin-stimulated whole body glucose turnover was estimated with a continuous infusion of D-[3-^3^H]glucose (PerkinElmer Life and Analytical Sciences, Inc., Waltham, MA) for 2 h prior to the clamps (0.05 µCi/min) and throughout the clamps (0.1 µCi/min). Insulin-stimulated whole body glucose uptake was calculated as the ratio of the [^3^H]glucose infusion rate to the specific activity of the plasma glucose during the final 30 min. of the clamps. Whole body glycolysis was calculated from the rate of increase in the concentration of ^3^H_2_O in plasma from minutes 90–120 of the clamps. Insulin-stimulated whole body glycogen plus lipid synthesis was estimated by subtracting whole body glycolysis from whole body glucose uptake. Blood samples were taken before, during, and at the end of the clamps to determine the concentrations of plasma [^3^H]glucose, ^3^H_2_O, 2-[^14^C]DG and insulin. At the end of the clamps, tissues were harvested for biochemical analysis as described previously [28].

### Liver and Muscle Glycogen

Tissues were collected and frozen on dry ice and stored at −80°C. 100 mg of liver or muscle was homogenized in 1 mL of homogenization buffer (20 mM Tris-HCl pH 7.5, 150 mM NaCl, 25 mM β-glycerophosphate, 20 mM sodium fluoride, 1 mM sodium orthovanadate, 2 mM sodium pyrophosphate, 2 mM EDTA, and Roche Complete protease inhibitor cocktail) using a TissueLyser II (Qiagen, Valencia, CA). The lysates were centrifuged at 14,000 rpm for 15 min at 4°C. The supernatants were aliquoted and stored at −80°C. 3 µl of tissue lysate supernatant was diluted with 147 µl water and then dried under vacuum in a Savant speed vac without heat. Trifluoroacetic acid (200 µl of 4 N) was added and incubated at 100°C for 4 h. The samples were centrifuged and dried under vacuum without heat. The dried samples were dissolved in 18 µM 2-deoxy-D-glucose and then analyzed with HPAE-PAD on a Dionex BioLC with a CarboPac PA10 column (isocratic elution with 20% 200 mM NaOH, 80% water) coupled to an ED40 electrochemical detector.

### Statistical analysis

Data were analyzed by the one-way ANOVA followed by Boneferroni's post-hoc test using the Prism 4 software program (GraphPad Software, Inc., San Diego, CA). Data were considered significant if P<0.05.
